# CMTM4 is frequently downregulated and functions as a tumour suppressor in clear cell renal cell carcinoma

**DOI:** 10.1186/s13046-015-0236-4

**Published:** 2015-10-16

**Authors:** Ting Li, Yingying Cheng, Pingzhang Wang, Wenyan Wang, Fengzhan Hu, Xiaoning Mo, Hongxia Lv, Tao Xu, Wenling Han

**Affiliations:** Peking University Center for Human Disease Genomics, Department of Immunology, Key Laboratory of Medical Immunology, Ministry of Health, School of Basic Medical Sciences, Peking University Center for Human Disease Genomics, Peking University Health Science Center, 38 Xueyuan Road, Beijing, 100191 China; Department of Urology, Peking University People’s Hospital, 11 Xi-Zhi-Men South Street, Beijing, 100044 China

**Keywords:** CMTM4, Clear cell renal cell carcinoma, Brain cancer, Tumour suppressor gene, G2/M cell cycle arrest, p21

## Abstract

**Background:**

*Chemokine-like factor (CKLF)-like MARVEL transmembrane domain-containing family* (*CMTM*) is a gene family involved in multiple malignancies. *CMTM4* is a member of this family and is located at chromosome 16q22.1, a locus that harbours a number of tumour suppressor genes. It has been defined as a regulator of cell cycle and division in HeLa cells; however, its roles in tumourigenesis remain poorly studied.

**Methods:**

An integrated bioinformatics analysis based on the array data from the GEO database was conducted to view the differential expression of CMTM4 across multiple cancers and their corresponding control tissues. Primary clear cell renal cell carcinoma (ccRCC) and the paired adjacent non-tumour tissues were then collected to examine the expression of CMTM4 by western blotting, immunohistochemistry, and quantitative RT-PCR. The ccRCC cell lines A498 and 786-O and the normal renal tubular epithelial cell line HK-2 were also tested for CMTM4 expression by western blotting. Cell Counting Kit-8 (CCK-8) and viable cell counting assays were used to delineate the growth curves of 786-O cells after CMTM4 overexpression or knockdown. Wound healing and transwell assays were performed to assess the cells’ ability to migrate. The effects of CMTM4 on cellular apoptosis and cell cycle progression were analysed by flow cytometry, and cell cycle hallmarks were detected by western blotting and RT-PCR. The xenograft model in nude mice was used to elucidate the function of CMTM4 in tumourigenesis ex vivo.

**Results:**

By omic data analysis, we found a substantial downregulation of CMTM4 in ccRCC. Western blotting then confirmed that CMTM4 was dramatically reduced in 86.9 % (53/61) of ccRCC tissues compared with the paired adjacent non-tumour tissues, as well as in the 786-O and A498 ccRCC cell lines. Restoration of CMTM4 significantly suppressed 786-O cell growth by inducing G2/M cell cycle arrest and p21 upregulation, and cell migration was also inhibited. However, knockdown of CMTM4 led to a completely opposite effect on these cell behaviours. Overexpression of CMTM4 also markedly inhibited the tumour xenograft growth in nude mice.

**Conclusions:**

CMTM4 is downregulated and exhibits tumour-suppressor activities in ccRCC, and could be exploited as a target for ccRCC treatment.

## Background

Renal cell carcinoma (RCC) is the most prevalent malignancy of the kidney, and it accounts for 2.4 % of all adult malignancies [1]. Clear cell renal cell carcinoma (ccRCC) represents the predominant histologic subtype of RCC and constitutes approximately 80-90 % of all cases [1, 2]. Surgery is the most effective treatment of early and local ccRCCs, but after the resection for local disease, 20–40 % patients will develop recurrence [3], mainly due to the tumour’s high resistance to both chemotherapy and radiotherapy [2, 4]. Therefore, it is of paramount importance to understand the molecular mechanisms underlying the tumourigenesis of ccRCC. The identification of novel genes that are functionally involved in the initiation and progression of ccRCC may provide more sophisticated early diagnostic and further therapeutic strategies.

The human *chemokine-like factor* (*CKLF*)*-like MARVEL transmembrane domain-containing family* (*CMTM*) is a gene family consisting of nine members, *CKLF* and *CMTM1-8* [5, 6]. Their encoded products are structurally and functionally intermediate between classical chemokines and the transmembrane-4 superfamily (TM4SF), playing important roles in the immune system [7–11], the male reproductive system [12–14] and tumourigenesis [15–25]. Several members, such as *CMTM3*, *5*, *7* and *8,* have been reported to exhibit tumour suppressor functions in many types of malignancies, including gastric, pancreatic, liver, lung, cervical, oral, ovarian and oesophageal cancers [15–25].

*CMTM4* is the most conserved member of this family and forms a gene cluster with *CKLF* and *CMTM1-3* on chromosome 16q22.1, a locus that is frequently deleted or modified in multiple tumours and that harbours a number of tumour suppressor genes [26–33]. *CMTM4* encodes three transcript variants, CMTM4-v1, −v2 and -v3. Among them, CMTM4-v2 is the full length cDNA product and is highly conserved in most vertebrate animals [34]. In HeLa cells, knockdown of CMTM4 can lead to cell cleavage defects and binucleated cells after mitosis [35], while overexpression of CMTM4-v1 and -v2 can inhibit cell growth by causing G2/M phase arrest without inducing apoptosis [34]. These findings suggest that *CMTM4* might be an important gene involved in cell growth and cell cycle regulation. However, the function of CMTM4 in tumourigenesis remains poorly defined. In this study, we analysed the expression pattern of CMTM4 using a bioinformatics strategy and focused on its expression and function in ccRCC.

## Materials and methods

### Bioinformatics

All of the array data related to cancers from the Affymetrix human genome U133 plus 2.0 platform were downloaded from the GEO database (http://www.ncbi.nlm.nih.gov/geo/), and a TumourProfile database (http://tumour.bjmu.edu.cn/, unpublished) has been developed to analyse the differentially expressed genes in tumours using previously described data processing and microarray analysis methods [36, 37]. The expression profile of CMTM4 in a variety of cancers and the corresponding control (normal or non-tumour) tissues was searched in this database, and the expression levels were represented as average rank scores (ARS). Rank-based gene expression (RBE) curves, which visually reflected the gene expression profile (GEP) across multiple tissues, were generated using the TumourProfile data set.

### Patient samples

A total of 61 patients with ccRCC (aged 22 to 78 years, median age of 60 years) who underwent surgery between January 2013 and April 2014 at the Department of Urology, Peking University People’s Hospital (Beijing, China) were enrolled in the present study. Paired tumour and adjacent non-tumour tissues were collected and tested for CMTM4 expression. All of the specimens were pathologically confirmed. The paraffin-embedded blocks of tumour tissues from each patient were assembled from the archival collections at the Department of Pathology. All participants gave informed consent according to the Helsinki Declaration, and the protocol for the present study was approved by the Ethics Committee of Peking University People’s Hospital (Beijing, China).

### Cell lines, adenovirus and siRNAs

The ccRCC cell lines A498 and 786-O and the normal renal tubular epithelial cell line HK-2 were routinely cultured in MEM (Invitrogen, Carlsbad, CA, USA), RPMI-1640 (HyClone, Logan, UT), and K-SFM medium (Gibco™ Life Technologies, Grand Island, NY) containing 10 % FBS (HyClone) supplemented with 1 % penicillin/streptomycin, respectively. All cells were grown at 37 °C in a humidified incubator containing 5 % CO2. Adenoviruses carrying the CMTM4 gene (Ad-CMTM4) and the empty adenovirus (Ad-null) were packaged by AGTC Gene Technology Company, Ltd. (Beijing, China). The 786-O cells were infected with the adenoviruses at an MOI of 100. Small interfering RNAs (siRNAs) targeting CMTM4 were designed and chemically synthesised by GenePharma Co., Ltd. (Suzhou, China). The following sequences were used: si-CMTM4-3, 5′-GAAAUUGCUGCCGUGAUAUTT-3′ (sense), 5′-AUAUCACGGCAGCAAUUUCTT-3′ (antisense); si-CMTM4-6, 5′-GCAUAUGCAGUGAACACAUTT-3′ (sense), 5′-AUGUGUUCACUGCAUAUGCTT-3′ (antisense); and negative control (si-NC), 5′-UUCUCCGAACGUGUCACGUTT-3′ (sense), 5′-ACGUGACACGUUCGGAGAATT-3′ (antisense). 786-O cells were transfected with the siRNAs using Lipofectamine™ 3000 (Life Technologies, Grand Island, NY) according to the manufacturer’s instructions.

### Protein extraction and western blotting

The cells were lysed in RIPA buffer (Sigma-Aldrich, St. Louis, MO, USA) supplemented with a 1 % protease inhibitor cocktail (Roche, Basel, Switzerland). The protein concentrations were determined using BCA protein assays (Pierce, Rockford, IL, USA). The whole cell lysates were then fractionated using 12.5 % or 15 % SDS–PAGE gels and electrotransferred onto polyvinylidene difluoride membranes (Hybond; GE Healthcare, Buckinghamshire, United Kingdom). Western blotting was performed as previously described [18]. The rabbit anti-CMTM4 pAb was prepared in our lab [38]. The anti-cyclin B1, −cyclin E, −cyclin-D1, −p21 and -p27 were purchased from Santa Cruz Biotechnology (Santa Cruz, CA, USA). β-actin blotting was used as a lysate loading control. The density of the bands was analysed by ImageJ software (National Institutes of Health, Bethesda, Maryland, U.S.). The absolute intensity of the target protein was normalised to the absolute intensity of β-actin.

### PCR and qPCR

The total RNAs were isolated from ccRCC tissues and cell lines using TRIzol reagent (Invitrogen). Reverse transcription was performed according to standard protocols using a RevertAid™ II First Strand cDNA synthesis Kit (Thermo Fisher Scientific Inc., Waltham, MA USA). Semiquantitative and quantitative PCR (qPCR) were performed as previously described [18]. GAPDH was amplified as an internal standard. The primers for PCR of CMTM4 were as follows: CMTM4V2-F: 5′-CAGAAATTGCTGCCGTGAT-3′, CMTM4V2-R: 5′-TGACTGAGAGACAGGCACG-3′, and the 72# probe (Roche) was used for qRT-PCR of CMTM4. The primers for PCR of p21 were p21-F: 5′-CTCAGAGGAGGCGCCATGTC-3′ and P21-R: 5′-TTAGGGCTTCCTCTTGGAGAAG-3′.

### Immunohistochemistry (IHC)

Immunohistochemical analysis was performed on formalin-fixed, paraffin-embedded clinical tissues as previously described [18]. A rabbit anti-CMTM4 pAb (4 mg/L) was used as the primary antibody.

### Cell proliferation assay

Cell proliferation was analysed using the Cell Counting Kit-8 (CCK-8, Dojindo Laboratories, Kumamoto, Japan) and viable cell counting assays. For the CCK-8 assays, the cells were seeded in 96-well plates at a density of 3000 cells per well and then incubated at 37 °C in a 5 % CO2 humidified atmosphere. At the indicated time points, 10 μL CCK-8 solution was added into each well and incubated for 2 h. The absorbance at 450 nm was measured to assess the number of viable cells. The results were obtained from three independent experiments in triplicate. For the viable cell counting assays, the cells were seeded in 24-well plates at a density of 20,000 cells per well. The viable cells marked by trypan blue exclusion were counted using a Vi-CELL TM_XR Cell Viability Analyzer (Beckman Coulter, Inc., Brea, CA, USA).

### Flow cytometry

Cellular apoptosis was evaluated by FITC-conjugated Annexin V/propidium iodide (PI) staining followed by flow cytometry analysis, as previously described [18]. For the cell cycle analysis, the cells were harvested 48 h after infection with adenoviruses or transfection with siRNAs. After washing with PBS, the cells were fixed in ice-cold 70 % ethanol overnight at −20 °C. The fixed cells were then pelleted by centrifugation, washed twice in PBS, and incubated in PBS containing 500 mg/mL RNase A (Sigma-Aldrich) at 37 °C for 30 min. After staining with 10 mg/mL PI (Sigma-Aldrich) in 0.1 % Triton X-100, the cells were collected on a BD FACSCalibur (BD Bioscience, San Jose, CA, USA). The cell cycle distribution was analysed with the ModFit LT software (Verity Software House, Topsham, ME).

### Wound healing assay

The 786-O cells infected with Ad-CMTM4 or Ad-null were cultured in 24-well plates until confluent. The cell layer was then scratched using a sterile 10 μL micropipette tip and washed twice with and subsequently maintained in serum-free media. The cells were photographed 0, 24 and 48 h after wounding.

### Cell migration assay

Forty-eight hours after infection or transfection, the 786-O cells were serum-starved for 6 h. Then, 3 × 10^4^ cells in 250 μL serum-free media were seeded into the upper chamber of a transwell with a fibronectin-coated filter (8-mm pore size, Corning Life Sciences, NY, USA). The bottom chamber contained medium supplemented with 10 % FBS. After a 14-h (for the siRNA-transfected cells) or 16-h (for the adenovirus-infected cells) incubation at 37 °C in a 5 % CO2 humidified atmosphere, the nonmigrated cells were scraped off of the filter using a cotton swab and the migrated cells were stained with crystal violet following fixation with 4 % paraformaldehyde. The number of cells was counted in 8 randomly chosen fields (magnification, ×200). Triplicate wells were performed in each assay, and the assay was repeated at least three times.

### Xenograft model in nude mice

All protocols for the animal studies were reviewed and approved by the institutional Animal Research Ethics Board. Female BALB/c nude mice (4–6 weeks old, weighing 18–22 g) were maintained in a germ-free environment in the animal facility. The tumourigenesis assay was performed as previously described, with some modifications [39]. Briefly, 5 × 10^6^ Ad-CMTM4- or Ad-null-infected 786-O cells in 100 μL PBS were injected subcutaneously into the right and left flanks of nude mice, respectively. The tumour diameter was measured with a calliper every 3 days, and the tumour volume was calculated by length × width^2^ × 0.5. The mice were sacrificed at day 27, when the tumours were dissected, weighed and lysed for western blotting analysis.

### Statistical analysis

The bioinformatics analysis of the differences in CMTM4 expression between the cancers and control tissues were evaluated using the Wilcoxon rank-sum test in the R (http://www.r-project.org/) software environment. Bonferroni’s correction of the R function “p.adjust” was used to adjust the *P*-values. The experimental data were analysed using SPSS software 17.0 (SPSS, Inc., Chicago, IL, USA). CMTM4 expression was correlated with the clinical characteristics using one-way ANOVA (for the classification variables, such as gender, stage and grade) or Pearson’s correlation analysis for two variables (for the continuous variables, such as age). The differences between two independent groups were analysed using Student’s t test. A *P*-value < 0.05 was considered to represent a statistically significant difference.

## Results

### CMTM4 is downregulated in ccRCC and brain cancers according to the omic data analysis

**Fig. 1:**
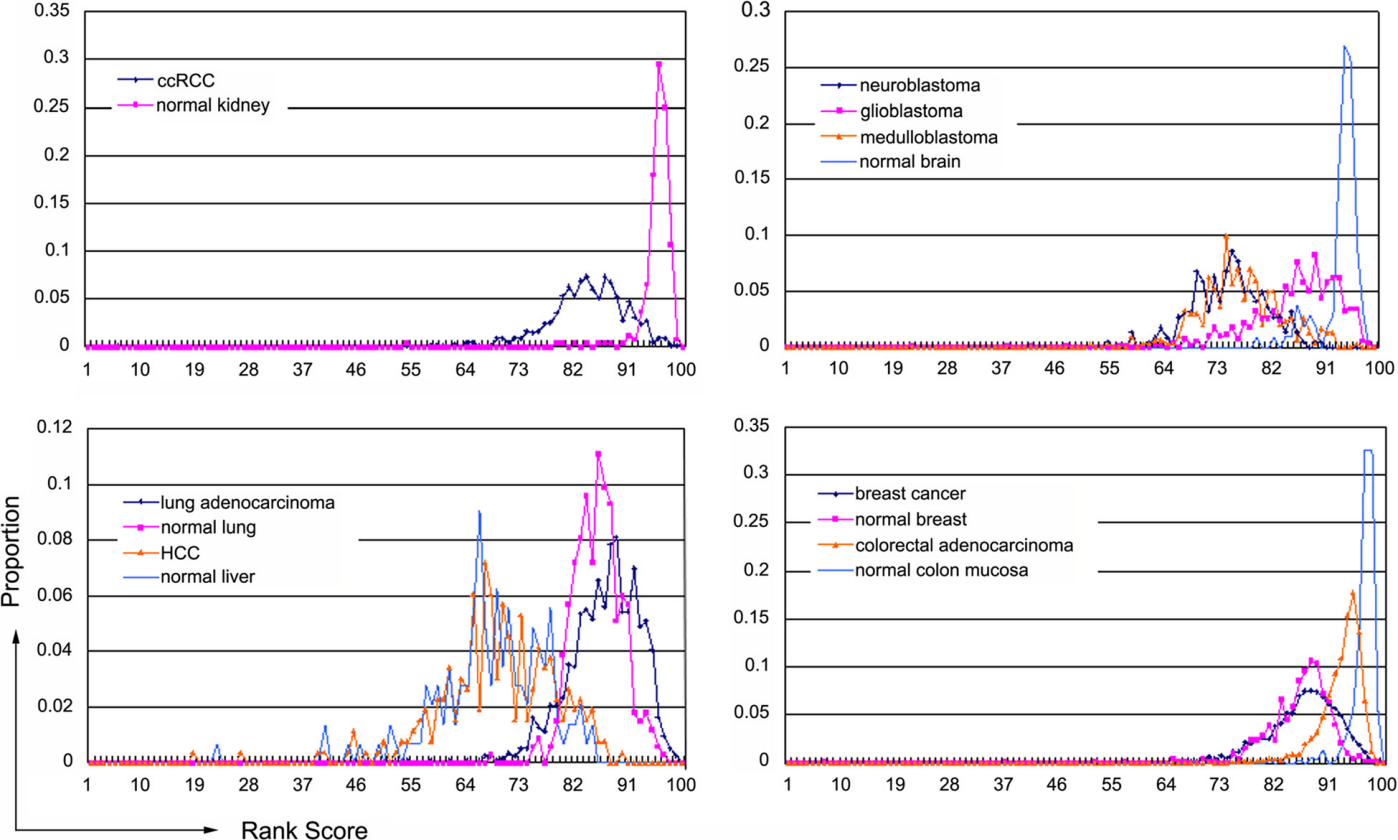
CMTM4 is downregulated at the mRNA level in ccRCC and brain cancers, according to bioinformatic analysis. The probe set 224998_at was used. In the rank-based gene expression (RBE) curves, the x-axis represents the expression intensity reflected by the rank scores, and the y-axis indicates the sample percentiles at each rank score

Gene expression profiles can reveal essential clues regarding a gene’s function. To assess the potential of CMTM4 as a tumour suppressor, we performed an integrated bioinformatics analysis based on the omic tumour data set from the GEO database to determine the differential expression of CMTM4 across multiple cancers and their corresponding control (normal or non-tumour) tissues at the mRNA level. The average rank scores (ARS), Bonferroni correction adjusted *P*-values (Table 1), and rank-based gene expression (RBE) curves (Fig. 1) were synthesised, and CMTM4 was most significantly downregulated in ccRCC and several brain cancers, such as neuroblastoma, glioblastoma, and medulloblastoma, while no apparent differences were observed in breast cancers, lung adenocarcinomas, hepatocellular carcinomas (HCCs), etc. The downregulation of CMTM4 in glioblastoma has been verified by a recent study [25], and that in ccRCC is further supported by the BioXpress (http://hive.biochemistry.gwu.edu/tools/bioxpress) [40] and the protein atlas (http://www.proteinatlas.org/ENSG00000183723-CMTM4/cancer) databases. The BioXpress database indicates that CMTM4 is downregulated in 95.83 % of ccRCC samples compared with their paired normal samples based on RNA sequencing (RNA-seq); the data set deposited in the Cancer Genome Atlas (TCGA) from a total of 128 patients has been collected and used for the analysis [40]. The protein atlas indicates that the CMTM4 protein is also expressed at lower levels in renal cancer tissues (*n* = 12, in general, weakly stained or negative) than in normal kidney tissues (*n* = 2, moderately positive).

**Table 1:** The average expression intensities of CMTM4 in multiple cancers

Tissue	Sample size	ARS ^a^	*P*-value ^b^	Bonferroni ^c^
bladder cancer	186	76.85	0.0068754	1
bladder control	64	68.66		
astrocytoma	207	89.69	1.68E-12	9.18E-08
ependymoma	152	94.47	0.0024976	1
glioblastoma ^d^	498	86.55	3.01E-28	1.65E-23
medulloblastoma ^d^	302	77.27	8.22E-49	4.48E-44
meningioma ^d^	89	85.75	1.69E-24	9.24E-20
neuroblastoma ^d^	221	75.38	5.83E-47	3.18E-42
oligodendroglioma	117	87.55	7.10E-07	0.038745
retinoblastoma ^d^	78	71.50	7.52E-29	4.11E-24
brain control	104	94.09		
breast cancer	3080	86.27	0.0209644	1
breast control	374	85.54		
colorectal adenocarcinoma	1841	92.80	3.59E-91	1.96E-86
colon mucosa normal	273	96.33		
gastric cancer	681	87.53	9.01E-13	4.91E-08
gastric control	61	92.61		
head and neck squamous cell carcinoma	220	80.40	0.0990636	1
head and neck squamous cell control	59	83.71		
ccRCC ^d^	652	83.83	1.58E-100	8.60E-96
kidney control	244	95.42		
hepatocellular carcinoma	263	68.85	0.4185167	1
liver control	62	69.47		
lung adenocarcinoma	1158	86.85	2.46E-06	0.1344489
lung squamous cell carcinoma	422	82.11	1.11E-15	6.09E-11
lung control	333	85.84		
oral squamous cell carcinoma	339	80.12	0.0026408	1
oral squamous cell control	118	81.62		
ovarian cancer	379	86.87	2.78E-05	1
ovary control	120	89.28		
pancreatic cancer	263	84.44	0.0205749	1
pancreas control	83	86.73		
prostate cancer	345	86.29	6.11E-12	3.33E-07
prostate control	81	72.88		
skin melanoma	522	79.30	6.76E-15	3.69E-10
skin normal	305	84.12		

^a^ARS denotes the average rank score

^b^The *P*-values were calculated using the Wilcoxon rank-sum test in the R (http://www.r-project.org/) software environment and are relative to the corresponding normal or non-tumour tissues

^c^The *P*-values were adjusted using Bonferroni correction in the function “p.adjust” in the R software

^d^The expression levels of CMTM4 in the underlined tissues were considered to be significantly downregulated by fully considering the differences in the ARS values, the Bonferroni correction adjusted P-values and the RBE curves shown in Fig. 1

### CMTM4 is frequently reduced in ccRCC tissues and cell lines

**Fig. 2:**
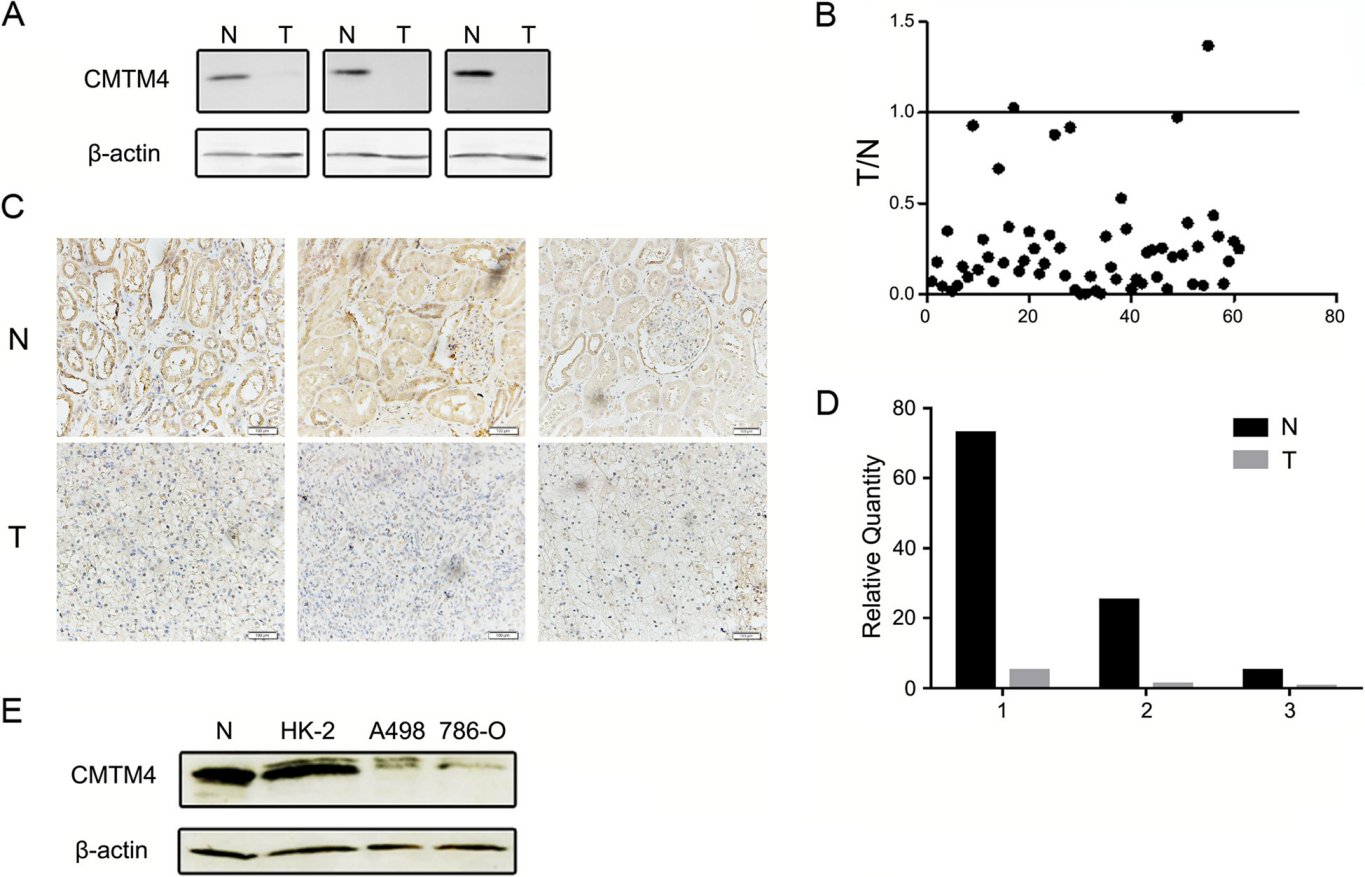
CMTM4 is frequently downregulated in ccRCC tissues and cell lines. **a** CMTM4 expression in the tumour (T) and paired adjacent non-tumour (N) tissues was detected by western blotting. The results of three representative paired tissues are shown. β-actin was used as an internal standard. **b** The relative CMTM4 expression level in all 61 patients was calculated for the tumour versus paired adjacent non-tumour tissue (T/N) according to the western blotting result and represented as a dot for each sample. CMTM4 was considered to be significantly downregulated only if the calculated fold change was less than 0.5 in the tumour tissue compared to the matched adjacent non-tumour tissue. CMTM4 expression was further examined by immunohistochemistry (**c**) and qRT-PCR (**d**) in representative samples. For qRT-PCR the CMTM4 expression level in the 2# tumour tissue was set as 1, and GAPDH was used as the internal control. **e** CMTM4 expression in the normal renal tubular epithelial line HK-2 and the ccRCC cell lines 786-O and A498 was detected by western blotting. β-actin was used as an internal standard. N, normal kidney tissue

**Fig. 3:**
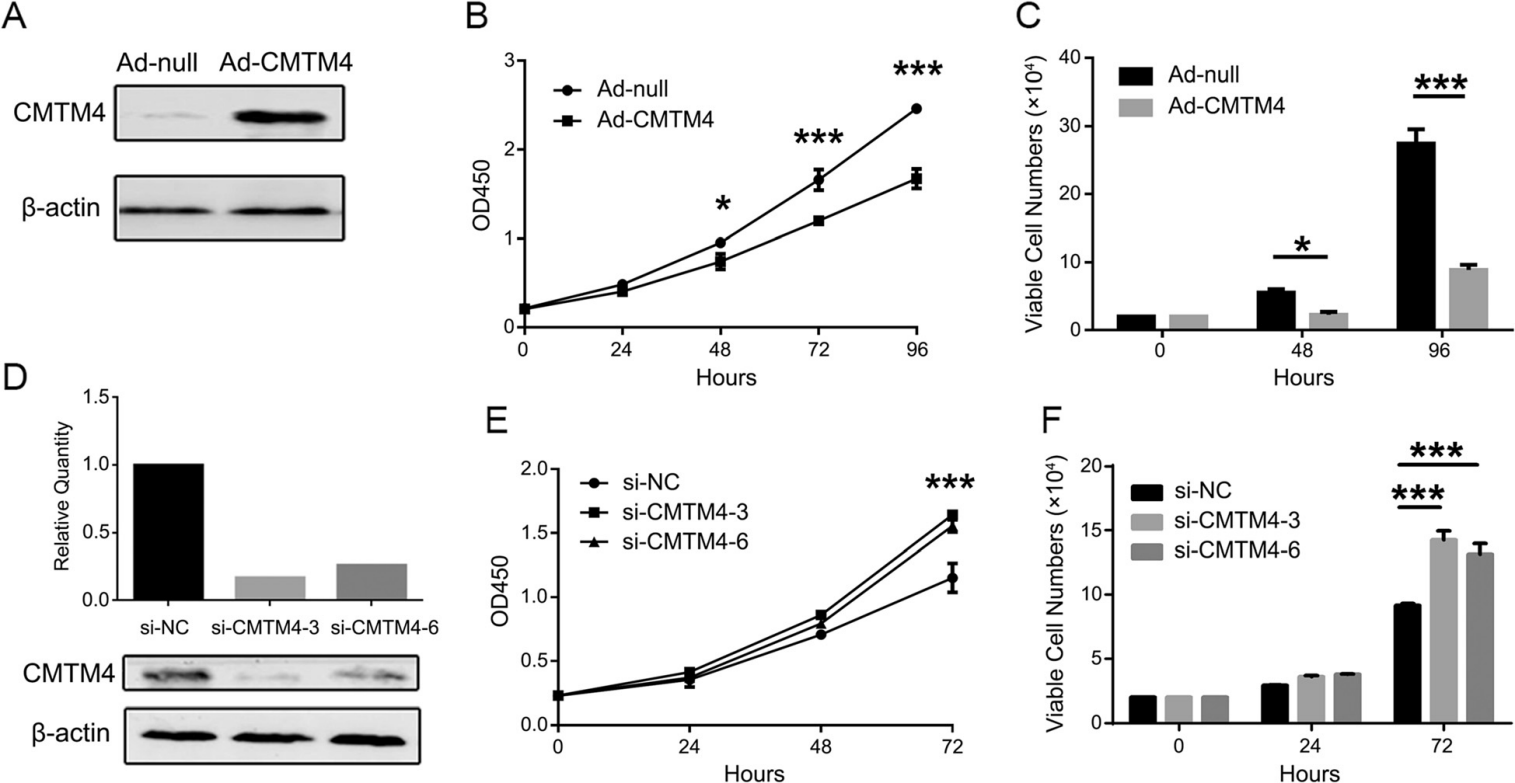
CMTM4 inhibits 786-O cell growth. **a** Overexpression of CMTM4 by infection with a CMTM4-expressing adenovirus (Ad-CMTM4) was confirmed by western blotting. The empty adenovirus (Ad-null) was used as a control. The Cell Counting Kit-8 (**b**) and viable cell counting (**c**) assays showed that cell growth was inhibited by overexpression of CMTM4 in 786-O cells. The results are expressed as the means ± SEM of three independent experiments in triplicate. *, *P* < 0.05; **, *P* < 0.01; and ***, *P* < 0.001 compared to the controls at each time point. **d** Knockdown of CMTM4 with two siRNAs (si-CMTM4-3 and 6) in 786-O cells was verified by quantitative RT-PCR (upper panel) and western blotting (lower panel). si-NC, negative control siRNA. The CMTM4 expression levels were measured relative to the level in the si-NC transfected cells in quantitative RT-PCR. The Cell Counting Kit-8 (**e**) and viable cell counting (**f**) assays showed that cell growth was promoted by knockdown of CMTM4 in 786-O cells. The results are expressed as the means ± SEM of three independent experiments in triplicate

According to the bioinformatics analysis, we then examined CMTM4 expression in 61 paired ccRCC tissues and adjacent normal tissues by western blotting. Compared to the non-tumour tissue, CMTM4 was dramatically downregulated in the ccRCC tissue. As the bands detected in western blotting were identical to those for the overexpressed CMTM4-v2 (~24 kDa, Fig. 3a), we focused on CMTM4-v2 in the subsequent studies, and the term “CMTM4” was used to indicate “CMTM4-v2”. The western blotting results of three representative paired tissues are shown in Fig. 2a, and quantitative analysis of the western blotting results of all 61 paired tissues was performed by normalizing the band density of CMTM4 to β-actin. The relative CMTM4 expression level was calculated for the tumour versus paired adjacent non-tumour tissue. As shown in Fig. 2b, the expression of CMTM4 was frequently downregulated in ccRCC tissues (53/61, 86.9 %) compared to the matched adjacent non-tumour tissues. We further analysed CMTM4 expression in representative samples by immunohistochemistry and qRT-PCR and obtained consistent results (Fig. 2c and d). Likewise, CMTM4 expression was also significantly lower in the ccRCC cell lines (786-O and A498) than in the normal renal tubular epithelial line HK-2 by western blotting (Fig. 2e).

### Correlations between CMTM4 expression and the clinical features

We also analysed the association between multiple clinical features of ccRCC patients and the expression of CMTM4 and observed no correlation between the CMTM4 expression levels and the parameters, including age, gender, clinical stage, and histologic grade (Table 2).

**Table 2:** Correlations between CMTM4 expression and the clinical features of the ccRCC patients

Clinical factors	Sample size	Mean ratio, T/N ± SEM ^a^	Correlation coefficient	*P* –value ^b^
Gender				0.863
Male	39	0.290 ± 0.318		
Female	22	0.223 ± 0.233		
Age	61		−0.022	0.864
Stage ^c^				n.a. ^d^
I	50			
I I	3			
III	5			
IV	3			
Grade				
1	27	0.182 ± 0.039		0.127
2	26	0.282 ± 0.050		n.a. ^d^
3	2	0.567 ± 0.313		

^a^The relative CMTM4 expression level was calculated from the tumour (T) versus paired adjacent non-tumour tissue (*N*)

^b^The *P*-value was calculated using one-way ANOVA (for the classification variables, such as gender, stage and grade) or Pearson correlation analysis for two variables (for the continuous variables, such as age)

^c^n.a. indicates not available due to a small sample size

^d^The tumour stage was defined according to TNM (International Union Against Cancer, 6th edition, 2002)

### CMTM4 inhibits 786-O cell growth

The reduced expression of CMTM4 in ccRCC prompted us to determine whether it plays an inhibitory role in tumourigenesis. The 786-O cells, in which CMTM4 was expressed at low levels, were infected with a CMTM4-expressing or empty adenovirus (Ad-CMTM4 or Ad-null), and cell growth was monitored over a 96-h period. The overexpression of CMTM4 was detected by western blotting (Fig. 3a). The CCK-8 (Fig. 3b) and viable cell counting (Fig. 3c) assays showed that CMTM4 significantly inhibited the proliferation of 786-O cells compared with the Ad-null infectants. Consistently with this finding, knockdown of CMTM4 with two siRNAs (si-CMTM4-3 and 6) in 786-O cells (Fig. 3d) was more potent than the negative control (si-NC) in promoting cell growth (Fig. 3e and f).

### CMTM4 causes G2/M cell cycle arrest

**Fig. 4:**
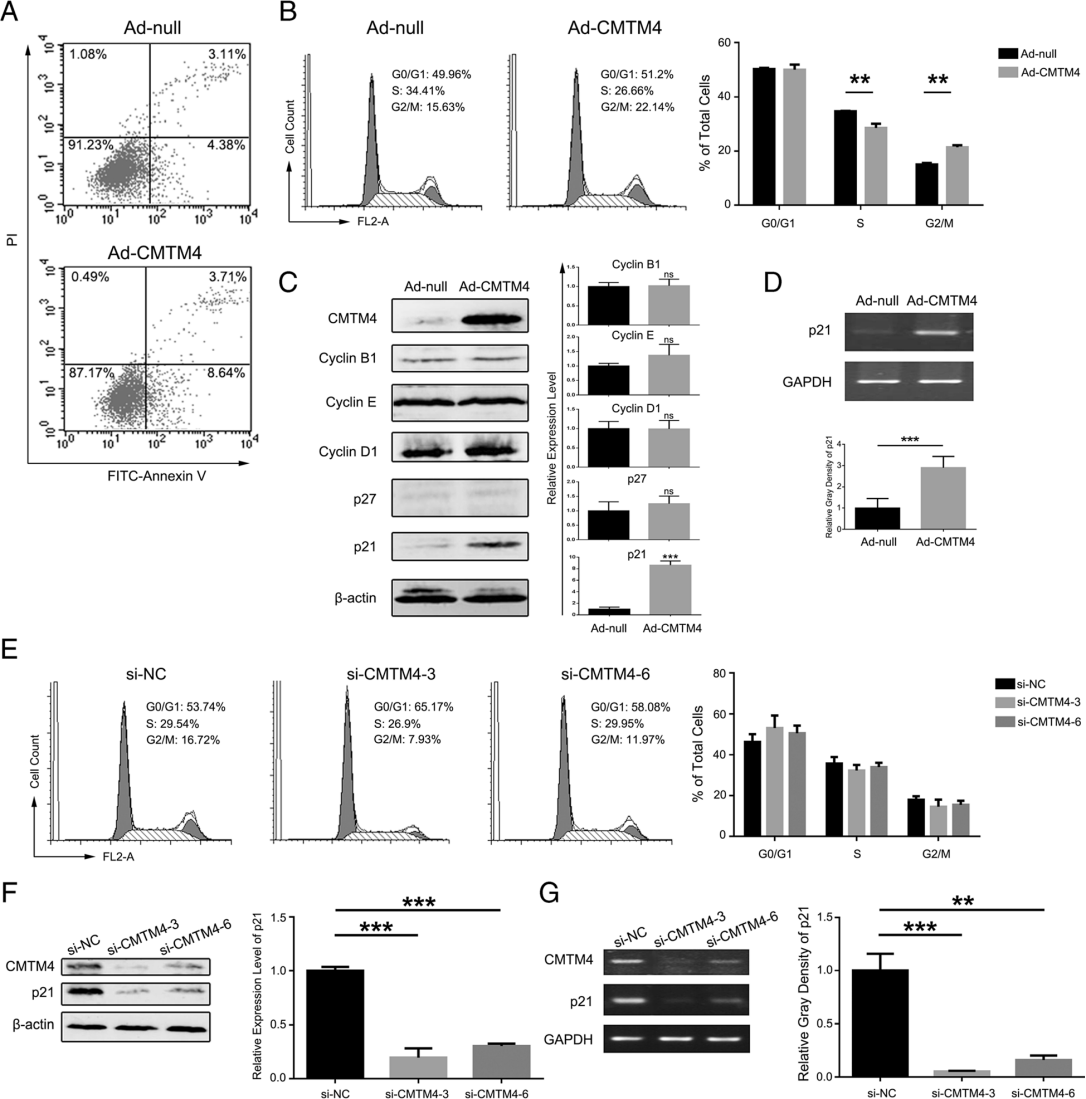
CMTM4 induces G2/M cell cycle arrest. **a** Annexin V/PI-staining indicated that overexpression of CMTM4 did not induce apoptosis of 786-O cells 72 h after infection. Shown is a representative result of three independent experiments. **b** The cell cycle was analysed 48 h after infection of 786-O cells by flow cytometry. Representative histograms (*left*) and the percentage of cells at the different phases (*right*) are shown. The data are expressed as the means ± SEM of three independent experiments. **, *P* < 0.01. **c** Western blotting analysis of cell cycle hallmarks in infected 786-O cells. β-actin was used as an internal standard. A representative result and the means of the relative intensities of the target proteins averaged for the three independent experiments are shown with the SEM. ***, *P* < 0.001; ns, not significant. **d** RT-PCR of p21 at 24 h after infection of 786-O cells. GAPDH was used as an internal standard. The grey density of the target bands was analysed by ImageJ software (National Institutes of Health, Bethesda, Maryland, U.S.) and normalised to the grey density of GAPDH. The average relative grey density with the SEM is shown from three independent experiments. Knockdown of CMTM4 reduced the G2/M phase accumulation (**e**) and p21 expression at both the protein (**f**) and mRNA levels (**g**). **, *P* < 0.01; and ***, *P* < 0.001

To elucidate the mechanisms underlying the tumour cell growth inhibition by CMTM4, its effects on apoptosis and cell cycle progression were studied by flow cytometry. FITC-Annexin V/PI staining indicated that overexpression of CMTM4 did not induce apoptosis of 786-O cells 72 h after infection (Fig. 4a). However, the Ad-CMTM4-infected cells had a significant increase in the G2/M phase population compared with the Ad-null infectants (Fig. 4b). We further examined several key cell cycle regulators by western blotting and found that p21 expression was upregulated in the CMTM4-expressing 786-O cells compared with the controls, whereas p27 and Cyclin B1, E and D1 were unaffected (Fig. 4c). RT-PCR was then performed and demonstrated that p21 expression was also upregulated at the mRNA level (Fig. 4d). However, knockdown of CMTM4 reduced the G2/M phase accumulation (Fig. 4e) and p21 expression at both the protein and mRNA levels (Fig. 4f and g). These results suggested that CMTM4 induces cell cycle arrest at the G2/M phase by upregulating p21 in 786-O cells.

### CMTM4 inhibits 786-O cell migration

**Fig. 5:**
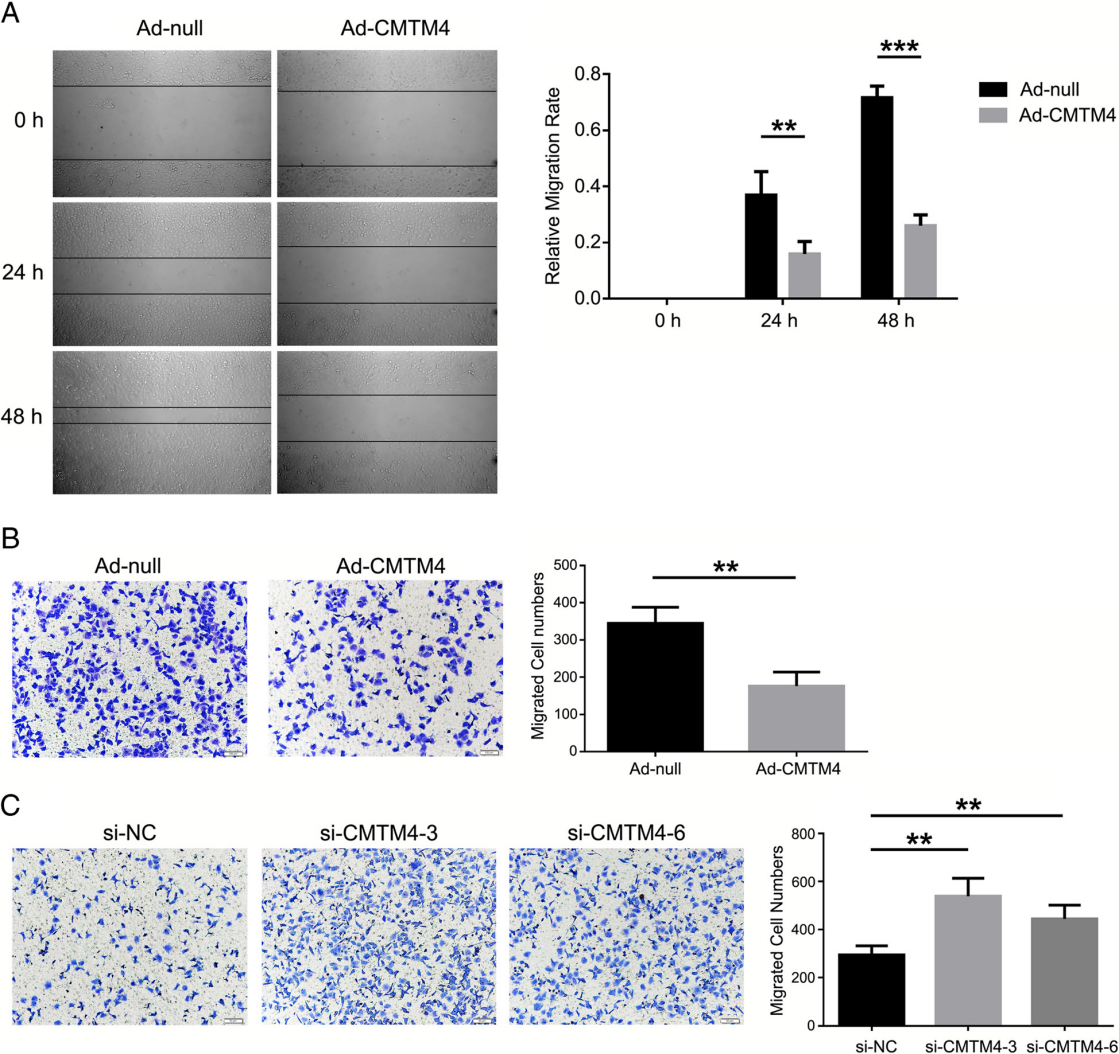
CMTM4 inhibits 786-O cell migration. **a** Representative images (magnification, ×100) of adenovirus-infected 786-O cell migration in the wound-healing assay were photographed at 0, 24 and 48 h after scratching (*left*). The relative migration rate was calculated by dividing the change in the distance between the scratch edges by the initial distance (*right*). Transwell assays (magnification, ×100) were performed to evaluate the migration of adenovirus-infected (**b**) and siRNAs-transfected (**c**) 786-O cells. The statistical graph indicates the means ± SEM of the number of cells from 8 random high power fields (magnification, ×200) counted from three independent experiments

Migration is another important aspect of tumourigenesis and has been reported to be negatively regulated by p21 [41]. We then explored the impact of CMTM4 on ccRCC cell migration. Wound-healing assays were first performed, and wound closure was found to be retarded for CMTM4-overexpressing 786-O cells (Fig. 5a). Transwell assays were then conducted to evaluate the motility of CMTM4 overexpressing or knockdown 786-O cells. Compared with their respective controls, overexpression of CMTM4 led to a significant decrease in the number of migrated cells (Fig. 5b), while knockdown of CMTM4 increased the number of 786-O cells that crossed over the filter (Fig. 5c).

### CMTM4 suppresses tumour growth ex vivo

**Fig. 6:**
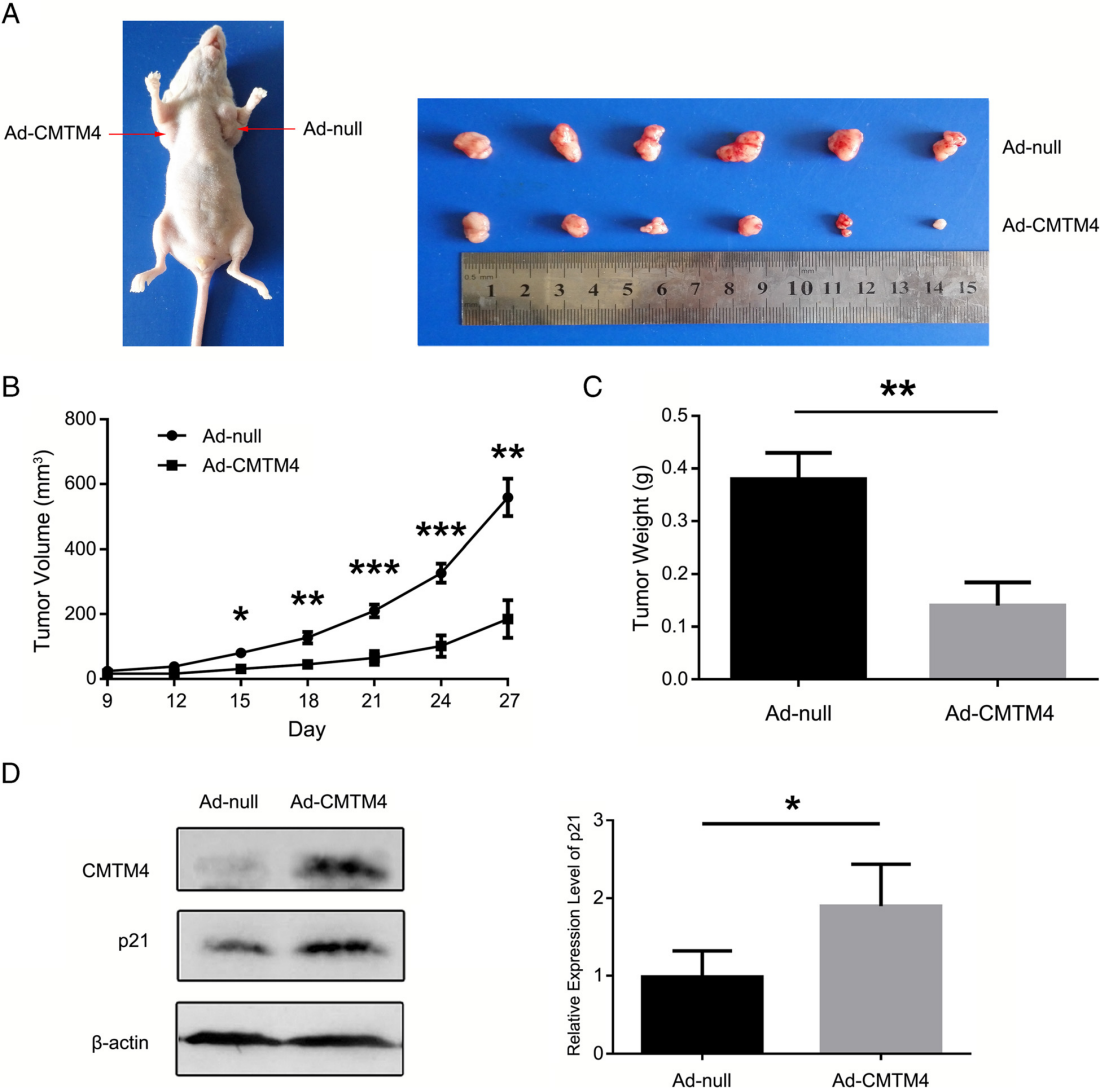
CMTM4 inhibits the tumourigenicity of 786-O cells ex vivo. **a** A representative nude mouse (left) showing the different morphologies of the tumours derived from the Ad-CMTM4-infected 786-O cells and Ad-null-infected control cells. The tumours were then dissected and photographed (*right*). The growth curve (**b**) and average weight (**c**) of the CMTM4-expressing tumours versus the control tumours are expressed as the means ± SEM for each group (*n* = 6). *, *P* < 0.05; **, *P* < 0.01; and ***, *P* < 0.001. **d** The expression of CMTM4 and p21 in the tumour xenografts was detected by western blotting. β-actin was used as an internal standard. A representative result of the tumours from one mouse (*left*) and the average relative intensity of p21 with the SEM from 6 mice (*right*) are shown. *, *P* < 0.05

The in vitro experiments demonstrated that CMTM4 exhibited antitumourigenic activities in ccRCC; therefore, we subsequently used a xenograft model in nude mice to confirm the ex vivo tumour-suppressor activity of CMTM4. 786-O cells infected with Ad-CMTM4 or Ad-null were injected subcutaneously into the right and left flanks of nude mice, respectively. The tumours appeared approximately one week after implantation. Within 4 weeks, the volume and weight of tumours from the CMTM4 overexpressing cells were significantly smaller than those of the controls (Fig. 6a-c). We also detected the expression of p21 in the tumour xenografts by western blotting and found that it was decreased in the CMTM4 overexpressing tumours (Fig. 6d). These data confirmed that CMTM4 exhibits tumour suppressor activities in ccRCC.

## Discussion

The tumour suppressor functions of members of the CMTM family, particularly CMTM3, 5, 7 and 8, have been extensively studied in multiple types of malignancies. In contrast, CMTM4 remains less investigated. A comprehensive analysis of CMTM4 expression across multiple cancers using bioinformatics indicated that CMTM4 is most significantly downregulated in brain cancers and ccRCC, which implies a tissue-specific function of CMTM4. Currently, omic data analysis has become a major trend in numerous fields, among which gene expression profile (GEP) analysis is generally an essential step in functional gene studies. Analyses using other databases, as well as Delic S. and colleagues’ [25] and our experimental data, demonstrate the viability of our analysis method [37] in GEP predictions, with high efficiency and accuracy.

Using a total of 61 paired ccRCC tissues and adjacent normal tissues, we show that CMTM4 expression is frequently downregulated in renal cancer tissues. However, the expression levels of CMTM4 were not correlated with the patients’ gender and age. Because surgical resection is restricted to early and local ccRCCs, most patients are at stage one and histologically exhibit high and moderate differentiation (grade I and II). Therefore, this correlation was not available due to the limitation of the clinical samples. Moreover, the survival data are still being collected, because most of the patients had undergone surgical resection only a short time ago.

*CMTM4* is tightly linked with *CMTM1-3* on chromosome 16q22.1, a genomic region prone to both genetic and epigenetic modifications in various cancers. Chromosomal aberrations, such as deletions, amplifications [26–29], single nucleotide polymorphisms (SNPs) [30], loss of heterozygosity (LOH) and microsatellite instability (MSI) [31, 32], as well as aberrant methylations [29], occur frequently in this region in different types of malignancies. Our previous studies have also shown that *CMTM3* is frequently inactivated by promoter CpG methylation [18]. It remains to be clarified whether these mechanisms are also involved in the downregulation of CMTM4 in ccRCC.

Regular cell cycle progression is a key factor in cell proliferation, and alterations of the cell cycle may influence cell growth. CMTM4 has been suggested to be an important regulator of cell cycle progression and division in HeLa cells [34, 35]. Here, we also observed that overexpression of CMTM4 inhibited 786-O cell growth by inducing G2/M phase accumulation. p21 was increased in the process, which plays complex roles in tumourigenesis by regulating the cell cycle, senescence, apoptosis and migration [41]. Through its interaction with the Cdk1/CyclinB complex, the p21 protein interferes with the transition of cells from the G2 phase of the cell cycle into mitosis; moreover, by inhibiting the Rho cascade, p21 can also influence cytoskeletal factors and cell motility [41]. Therefore, the upregulation of p21 may be responsible for the tumour suppressor functions of CMTM4 in 786-O cells. However, increased p21 expression is not necessarily linked to growth arrest; thus, the sophisticated mechanism underlying the inhibitory activities of CMTM4 is still to be explored. On the other hand, p21 is well known to be induced by p53. In addition, several p53-independent pathways have also been identified [42]. Overexpression of CMTM4 increased p21 not only at the protein level but also at the mRNA level, whereas knockdown of CMTM4 decreased both. However, because p53 is inactive in 786-O cells [43], the mechanism by which CMTM4 regulates p21 and whether it influences the transcription or the degradation of the p21 mRNA requires further investigation.

## Conclusions

In summary, CMTM4 is frequently reduced in ccRCC tissues and cell lines, according to omic data analysis as well as our experimental data. Restoration of CMTM4 suppresses the tumourigenicity of 786-O cells both in vitro and ex vivo, whereas knockdown of CMTM4 led to promoting effects. These observations highlight the potential of CMTM4 as a tumour suppressor in ccRCC. A better understanding of the roles of CMTM4 in tumourigenesis may allow researchers to develop novel diagnostics and more effective treatment strategies for this malignancy.

## Abbreviations

CMTM: Chemokine-like factor (CKLF)-like MARVEL transmembrane domain-containing family
GEO: Gene Expression Omnibus
RCC: Renal cell carcinoma
ccRCC: Clear cell renal cell carcinoma
ARS: Average rank score
RBE: Rank-based gene expression
GEP: Gene expression profile
MOI: Multiplicity of infection
siRNAs: Small interfering RNAs
qPCR: Quantitative PCR
IHC: Immunohistochemistry
CCK8: Cell Counting Kit-8
PI: Propidium iodide
